# Acuity of asthma exacerbations in Alberta, Canada is increasing: a population-based study

**DOI:** 10.1186/s13223-024-00872-0

**Published:** 2024-02-12

**Authors:** Adil Adatia, Jalal Moolji, Imran Satia

**Affiliations:** 1 Division of Pulmonary Medicine, Department of Medicine, University of Alberta, Edmonton, AB Canada; 2 Alberta Respiratory Centre, Edmonton, AB Canada; 3 Department of Medicine, McMaster University, Hamilton, ON Canada

**Keywords:** Asthma, Epidemiology, Asthma exacerbations, Emergency department, Hospitalization, Critical care

## Abstract

**Background:**

Asthma is a common respiratory illness affecting 2.8 million Canadians, including 9.7% of Albertans. Prior studies showed a substantial decrease in ED visits for asthma in the decade preceding 2010, followed by a stabilization. This was attributed to improvements in the pharmacologic and non-pharmacologic treatments for asthma during that period followed by a balance between epidemiologic drivers and protective factors in the population.

**Methods:**

We assessed whether this trend continued in Alberta from 2010 to 2022 using population level data for the volume of daily ED visits, acuity of asthma exacerbations in the ED, and hospitalization rate.

**Results:**

The mean number of ED visits decreased from 4.5 to 2.2 per million persons per day, but the acuity of exacerbations and the proportion requiring hospitalization increased. The number of patients presenting with the highest level of acuity increased by over 300%, and the percentage of patients requiring hospitalization increased from 6.8 to 11.3%.

**Conclusion:**

Total ED visits for asthma exacerbations continues to decline in Alberta. The reasons for an increase in more severe exacerbations requires further attention.

**Supplementary Information:**

The online version contains supplementary material available at 10.1186/s13223-024-00872-0.

## Introduction

Asthma is one of the most common respiratory diseases globally. In 2020, 2.8 million Canadians were affected by the condition, and the prevalence appears to be increasing [1]. In the province of Alberta, the prevalence increased from 6.46% in 2019 to 9.70% in 2020 [2].

A characteristic feature of asthma is intermittent symptoms, which if severe and persistent can lead to an exacerbation requiring oral prednisone or increasing the frequency or strength of inhaler therapy. Some exacerbations can result in healthcare utilization, including emergency department (ED) care when severe or when alternate care settings are not available. Prior studies in Alberta and Canada demonstrated a marked reduction in ED visits and hospitalizations for asthma in the decade before 2010 [3, 4]. Thereafter, hospitalizations for asthma appeared to stabilize [4]. This was attributed to a “floor effect” in which the beneficial effects of medications and improved outpatient care were balanced against persistent social and biologic drivers of asthma in the population. Subsequent studies have shown that the quality of asthma care in the community has not kept pace with the rising prevalence of asthma [2]. In view of this finding, we sought to investigate whether the rate of severe exacerbations did experience a floor effect in the province of Alberta in the years following 2010 by analyzing several epidemiologic trends in conjunction.

The objectives of this study were: (1) to investigate the rates of emergency department (ED) visits for asthma from 2010 to 2022, (2) to evaluate trends in the acuity of asthma ED presentations over this period, and (3) to determine the number of asthma patients in the ED who required hospital admission over this period.

## Methods

### Study design and data source

This retrospective population-based study aimed to investigate the temporal trends in asthma exacerbations among adults in Alberta, Canada, from 2010 to 2022. We obtained healthcare data from the Government of Alberta Interactive Health Data Application. We collected data on emergency department (ED) presentations and hospital admissions due to asthma exacerbations stratified by sex. Asthma-related ED visits and hospitalizations are logged within the system using the primary discharge diagnosis ICD-10 code J49. Information regarding the acuity of asthma exacerbations (Canadian Triage and Acuity Scale, CTAS) was obtained from the Alberta Health Services aggregated administrative database. Originally developed in 1999, CTAS is a standardized assessment tool used in Canadian EDs for triage and patient flow analysis. It consists of a database of presenting complaints and special modifiers that can be applied to patients presenting to the ED. A scale from 1 to 5 is generated, which estimates the patient’s acuity level, with 1 representing the highest acuity and 5 the lowest acuity [5]. The data used were publicly available, and research ethics approval was thus not required.

We extracted annual data on the average rate of asthma-related ED presentations per 1 million persons and the average daily rate of hospital admissions for asthma exacerbations from 2010 to 2022. These rates were calculated separately for all adults, males, and females. To gain further insight into the acuity of asthma exacerbations, we collected data on triage acuity using the CTAS for the same time frame (2010–2022).

### Data analysis

Temporal trends and sex differences in the rate of ED visits, the average daily rate of asthma-related hospitalizations and CTAS were analyzed graphically. Simple linear regression was used to quantify the rate of change in ED visit volume and proportion needing admission in the years prior to the pandemic (2010–2019). In addition, we used change point analysis to identify any notable shifts in the temporal trends of asthma exacerbations from 2010 to 2019. Change point analysis is an unbiased statistical technique used to detect “steps” or breakpoints in time series data. The pruned exact linear time algorithm was implemented using the Bayesian information criterion penalty to prevent overfitting [6]. All analyses were conducted using R (version 4.3.1).

## Results

**Fig. 1:**
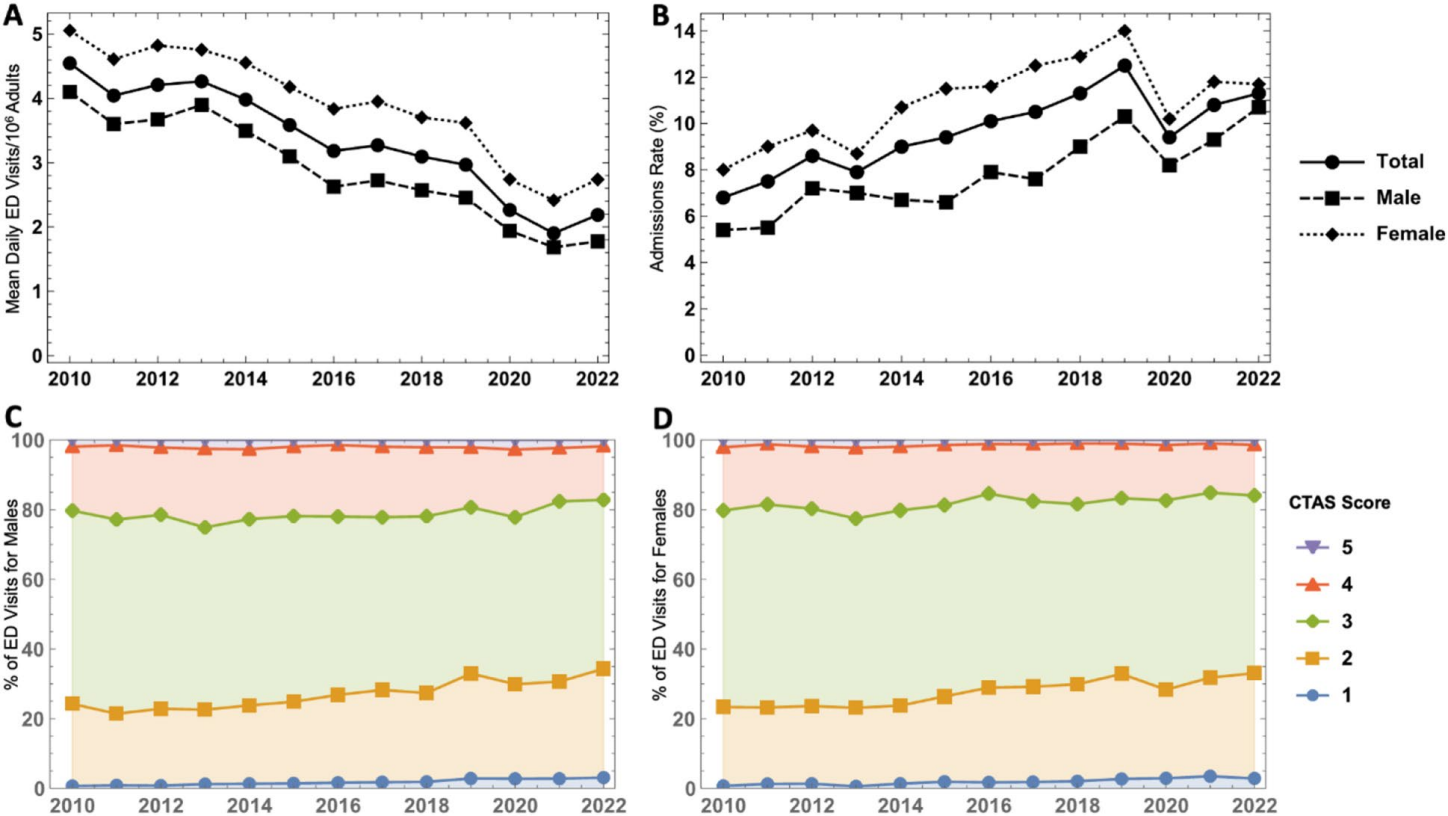
**A** Mean daily ED visits for asthma exacerbations per 10^6^ adults. **B** Percent of adult ED visits for asthma exacerbations resulting in hospital admission. **C** Stacked area plot showing proportion of ED visits for asthma exacerbations with each CTAS among males. **D** Stacked area plot showing proportion of ED visits for asthma exacerbations with each CTAS among females. The percentages in **C **and **D**. are additive to show changes in the distribution of CTAS scores over time and add to 100% for each year; Canadian Triage and Acuity Scale, *ED e*mergency department

The mean number of ED visits normalized by population size for asthma is shown in Fig. 1A. The province of Alberta’s estimated total adult population in 2010 was 2,919,822 and this increased by 22.6% to 3,578,279 in 2022. The number of ED visits declined by 34.7% from 2010 to 2019. There was a further decline with a nadir during the COVID-19 pandemic in 2021 at 1.90 visits per day/1 million adults.

The volume of ED visits was consistently higher among females compared to males. Over the years studied, the ED visit volume for females was on average 37.8% higher than that of males. Simple linear regression fit the ED visit volume prior to the COVID-19 pandemic (2010–2019) for males and females well (R^2^ = 0.94 for males and R^2^ = 0.91 for females). The rate of change of mean daily ED visits/1 million people was − 0.21 and − 0.20, in females and males, respectively.

The admissions rate for asthma exacerbations among adults is shown in Fig. 1B. The percent of ED visits for asthma that resulted in hospital admission increased from 6.8 in 2010 to the peak of 12.5% in 2019. The proportion of females presenting to the ED with asthma subsequently admitted was consistently higher compared to males. Simple linear regression fit the rate prior to the COVID-19 pandemic (2010–2019) for males and females well (R^2^ = 0.81 for males and R^2^ = 0.94 for females). The rate of change in admissions rate was 0.63 and 0.44% points per year, in females and males, respectively.

The CTAS for ED presentations for asthma are shown in Fig. 1C, D, for men and women respectively. Most presentations were coded as CTAS 3 and the proportion receiving this scale was similar across the period studied. There was an increase in the frequency of CTAS 1 and 2 presentations for both males and females. Between 2010 and 2022, the proportion of ED presentations coded as CTAS 1 increased from 0.66 to 3.09% in males (366% increase) and from 0.70 to 2.85% in females (305% increase). Over the same period, CTAS 2 ED presentations increased by 32.5% in males and 33.6% in females. There was an associated decrease in the frequency of CTAS 4 presentations.

Change point analysis did not identify any step changes in ED visit volume or admissions rate from 2010 to 2019.

## Discussion

We conducted a population study of ED visit volume, acuity, and hospital admission rates for asthma in Alberta, Canada. The primary findings of this study are: (1) the incidence of ED visits for asthma has decreased since 2010, (2) the acuity of asthma patients in the ED has simultaneously increased, and (3) the percent of ED visits for asthma resulting in hospital admission has increased.

This study builds on the work of Rosychuk et al. that looked at an earlier time period in Alberta, 1999–2011 [3]. They found that the number of ED visits for asthma in adults declined from 19.3 to 8.5 per million per day over this period. There was an associated decline in hospital admissions, from 10.3 to 6.9%. They hypothesized that better asthma therapeutics, non-pharmacological interventions, and health care access outside the ED were responsible for these improvements. This study demonstrates a continued, albeit gradual decline in ED visits, from 4.5 to 2.2 per million per day from 2010 to 2022. Unfortunately, the hospitalization trend has reversed, and there was a concomitant increase from 6.8 to 11.3%. The situation has become more complex as fewer asthmatics sought care in the ED, and those who did go to the ED were more acute and more likely to require hospitalization. Over the study period, the high-acuity ED visits (CTAS 1) increased by 3.7 fold in males and 3.1 fold in females.

It would be reassuring to state that fewer asthmatics presented to the ED because of better symptom control in the community, but that was probably not the case. Moitra et al. showed that the prescription of asthma controller inhalers in Alberta, including ICS, remained stable from 2008 to 2020 despite the increasing prevalence of asthma [2]. The Canadian obesity rate increased over the study period, asthmatics are more likely to develop obesity than the general population, and this would be associated with worse asthma outcomes [7–9]. The study period includes the legalization of cannabis in Canada in 2018, and the advent of electronic cigarettes. Forest fires have become more frequent and are associated with worse asthma control [10]. It is more likely that the number of poorly controlled asthmatics in the community has increased, and they have become less likely to visit the ED for mild or moderate exacerbations over the years. Multiple epidemiologic studies have theorized that ED overcrowding affects asthma patients’ decision to visit the ED [3, 4, 11]. This study suggests that patients who present to the ED regardless of overcrowding are more likely to have severe exacerbations and require hospitalization. This is supported by Alberta data suggesting that patients with the most severe exacerbations required increasing time in the ED for stabilization from 2011 to 2015 [12].

Lee et al. examined Canada-wide trends in asthma hospitalization from 2002–2017 [4]. They found a major improvement in hospitalization rates from 2002 to 2010, and a subsequent stabilization until 2017. They posited that advances in asthma therapeutics, like ICS/LABA, combined with non-pharmacologic approaches greatly improved asthma control until about 2010. Thereafter, there was a “floor effect,” whereby only a subset of asthmatics continued to have severe exacerbations, resulting in a stabilization of asthma hospitalizations. These patients may have had asthma endotypes that were poorly responsive to existing therapies, or experienced systemic and socioeconomic barriers to health care.

With regards to Alberta, there probably was no “floor effect,” as the gradual decline in ED visits masked a dynamic and worsening situation between 2010 and 2022. This and other studies have shown a worsening of the population prevalence of asthma, the acuity of exacerbations in the ED, the percentage of asthmatics in the ED requiring hospitalization, and non-prescription of controller medications in recent years [2].

However, there are reasons for optimism, particularly for patients with adequate access to specialist health care. Given the Covid-19 pandemic disrupted pre-existing trends in asthma, it is probably too early to assess the effectiveness of biologics for asthma using real-world epidemiologic data. Guideline-based approaches to the use of ICS/LABA on demand may result in improved symptom control and exacerbation rates. Additionally, the development of mRNA vaccines for common respiratory viruses, such as rhinovirus, may also reduce exacerbation rates.

There were sex differences in our data, with women showing consistently higher rates of ED visits and hospitalization than men. This is consistent with prior Canadian data showing that elderly women are less likely to have spirometry, physician visits for asthma, or controller medication prescription than elderly men [13, 14]. In Alberta, women presenting to the ED for asthma are more likely to return within 30 days than men [15].

There are limitations of this study. First, the reliance on administrative healthcare data may be subject to coding errors or biases. Second, although we assessed sex-specific trends, other demographic factors and clinical variables, such as age and co-morbidities, were not included in this analysis, and this may have influenced asthma exacerbation rates. Third, the unique circumstances of the Covid-19 pandemic, which did affect ED visit and hospitalization rates in our data, deserve special attention, and this is beyond the scope of our study. Other investigators have commented on the reduction in the number of severe asthma exacerbations during the pandemic, and speculated on the reasons Additional file 1.

## Conclusions

The rate of adult presentations to the emergency department for asthma gradually declined from 2010 to 2022 in Alberta, Canada. This may be related to patient avoidance of the emergency department due to overcrowding, except for the most severe exacerbations. Those presenting to the emergency department were increasingly severe over this period, as evidenced by higher acuity scales and higher rates of hospital admission. In the context of other studies on asthma epidemiology, we posit that the number of patients with uncontrolled asthma has increased in the community and asthma presentations to the ED have become increasingly severe over time.

### Supplementary Information

**Additional file 1: Table S1.** Number of asthma-related ED visits each year by CTAS score among males. **Table S2.** Number of asthma-related ED visits each year by CTAS score among females.

## Data Availability

All relevant data are contained within the manuscript.

## Abbreviations

CTAS: Canadian Triage and Acuity Scale
ICD: International Classification of Diseases
ED: Emergency department
