# Supplementary material for: Acuity of asthma exacerbations in Alberta, Canada is increasing: a population-based study
Source: Allergy Asthma Clin Immunol. 2024 Feb 12;20:13. doi: 10.1186/s13223-024-00872-0 (PMC10863092; doi:10.1186/s13223-024-00872-0)
Supplement: Supplementary file 1 — Additional file 1: Table S1. Number of asthma-related ED visits each year by CTAS score among males. Table S2. Number of asthma-related ED visits each year by CTAS score among females. [file 13223_2024_872_MOESM1_ESM.docx]

Table S1: Number of asthma-related ED visits each year by CTAS score among males

|  | 2010 | 2011 | 2012 | 2013 | 2014 | 2015 | 2016 | 2017 | 2018 | 2019 | 2020 | 2021 | 2022 |
| --- | --- | --- | --- | --- | --- | --- | --- | --- | --- | --- | --- | --- | --- |
| CTAS 1 | 11 | 17 | 16 | 27 | 27 | 26 | 25 | 28 | 29 | 42 | 32 | 26 | 34 |
| CTAS 2 | 391 | 396 | 452 | 474 | 461 | 427 | 390 | 422 | 394 | 448 | 315 | 261 | 344 |
| CTAS 3 | 919 | 1075 | 1139 | 1161 | 1096 | 971 | 792 | 790 | 784 | 709 | 556 | 485 | 533 |
| CTAS 4 | 304 | 410 | 395 | 500 | 410 | 363 | 317 | 322 | 306 | 255 | 225 | 143 | 169 |
| CTAS 5 | 32 | 30 | 45 | 57 | 55 | 35 | 23 | 31 | 33 | 32 | 32 | 22 | 20 |

Table S2: Number of asthma-related ED visits each year by CTAS score among females

|  | 2010 | 2011 | 2012 | 2013 | 2014 | 2015 | 2016 | 2017 | 2018 | 2019 | 2020 | 2021 | 2022 |
| --- | --- | --- | --- | --- | --- | --- | --- | --- | --- | --- | --- | --- | --- |
| CTAS 1 | 14 | 31 | 35 | 15 | 35 | 46 | 39 | 43 | 46 | 60 | 49 | 51 | 50 |
| CTAS 2 | 451 | 538 | 586 | 595 | 584 | 594 | 618 | 647 | 625 | 669 | 430 | 416 | 531 |
| CTAS 3 | 1125 | 1427 | 1490 | 1433 | 1462 | 1331 | 1264 | 1260 | 1161 | 1119 | 919 | 780 | 896 |
| CTAS 4 | 361 | 422 | 467 | 535 | 475 | 418 | 323 | 386 | 390 | 347 | 268 | 207 | 255 |
| CTAS 5 | 42 | 31 | 51 | 60 | 51 | 36 | 27 | 30 | 23 | 24 | 25 | 15 | 25 |
